# Effects of MP Polyethylene Microparticles on Microbiome and Inflammatory Response of Larval Zebrafish

**DOI:** 10.3390/toxics8030055

**Published:** 2020-08-11

**Authors:** Nicholas Kurchaba, Bryan J. Cassone, Caleb Northam, Bernadette F. Ardelli, Christophe M. R. LeMoine

**Affiliations:** Department of Biology, Brandon University, Brandon, MB R7A 6A9, Canada; kurchan90@brandonu.ca (N.K.); cassoneb@brandonu.ca (B.J.C.); northamc@myumanitoba.ca (C.N.); ardellib@brandonu.ca (B.F.A.)

**Keywords:** microplastic, dysbiosis, microbiome, freshwater

## Abstract

Plastic polymers have quickly become one of the most abundant materials on Earth due to their low production cost and high versatility. Unfortunately, some of the discarded plastic can make its way into the environment and become fragmented into smaller microscopic particles, termed secondary microplastics (MP). In addition, primary MP, purposely manufactured microscopic plastic particles, can also make their way into our environment via various routes. Owing to their size and resilience, these MP can then be easily ingested by living organisms. The effect of MP particles on living organisms is suspected to have negative implications, especially during early development. In this study, we examined the effects of polyethylene MP ingestion for four and ten days of exposure starting at 5 days post-fertilization (dpf). In particular, we examined the effects of polyethylene MP exposure on resting metabolic rate, on gene expression of several inflammatory and oxidative stress linked genes, and on microbiome composition between treatments. Overall, we found no evidence of broad metabolic disturbances or inflammatory markers in MP-exposed fish for either period of time. However, there was a significant increase in the oxidative stress mediator L-FABP that occurred at 15 dpf. Furthermore, the microbiome was disrupted by MP exposure, with evidence of an increased abundance of *Bacteroidetes* in MP fish, a combination frequently found in intestinal pathologies. Thus, it appears that acute polyethylene MP exposure can increase oxidative stress and dysbiosis, which may render the animal more susceptible to diseases.

## 1. Introduction

Plastic materials are inexpensively produced and provide a high level of durability and flexibility, making them a key material for various applications. While an increasing number of plastics are re-used and recycled, many “single use” plastics are readily discarded and accumulate as waste worldwide [1]. Over 4900 metric tons of plastic waste have been discarded in landfills and the environment since 1950 [1,2]. Discarded plastics are derived from different polymers, such as polyethylene, polypropylene, and polystyrene, with each polymer providing unique and desirable qualities. Polyethylene is particularly desirable for packaging due to its resistance to degradation and low economic cost and thus is of major environmental importance [3].

Plastic pollution affects most ecosystems, including freshwater and oceanic systems. In these environments, plastic litter undergoes photo-oxidation and physical breakdown, generating smaller and chemically stable particles. When these particles are smaller than 5 mm in diameter, they are called secondary microplastics (MP) and may persist extensively in the environment and in organisms [4,5,6,7]. In contrast, primary MP are intentionally manufactured for various applications [6]. Regardless of their provenance, primary and secondary MP have been found in oceans for decades, though more recently they have been detected in freshwater, reaching some of the most remote lakes around the world [2,8,9]. In freshwater reserves, such as the Ottawa River and the Nantaizi Lake, MP can attain concentrations between 0.1 and 6162.5 p/L [10,11,12]. As MP can easily be ingested, their presence in aquatic environments poses a potential risk to many animal species, chronically exposing them to this pervasive pollutant. In aquatic animals, MP primarily accumulate both in the alimentary canal and respiratory structures in adult animals, though they appear to be limited to the intestine in aquatic larvae [13,14].

Once ingested, MP can negatively impact organismal health at different levels [15,16,17]. First, MP can disrupt intestinal transit and their accumulation may trigger intestinal villi swelling [18]. In addition, at the cellular level, MP can also cause physical injury to surrounding cells via abrasion-induced oxidative stress and accumulation of intercellular reactive oxygen species (ROS) [19]. Enhanced intestinal ROS production may also indicate an inflammatory response and participate in a feedback loop [20]. Regardless of the cause, excess ROS production jeopardizes tissue integrity and overall organismal health [21]. 

The inflammatory response is coordinated by multiple cascades, including pro- and anti-inflammatory mediators. These cascades can be initiated by multiple stressors, such as toxins, tissue damage, or microorganisms [22], and may trigger cytokines to flood surrounding tissues and recruit nearby immune cells to initiate inflammation. While inflammation allows for the removal of the foreign stimuli and promotion of active healing of the injured area, prolonged inflammation can also result in various pathologies, such as inflammatory bowel disease, leading to metabolic dysfunction and nutrient deficiency [23,24].

The intestinal accumulation of MP may also affect its microbiome, another crucial player in animal wellbeing. In many organisms, including fish, it is clear that the microbiome plays a crucial role in both health and diseases; thus, disturbances in the gut microbiome can be detrimental to overall animal health [25,26,27]. As in other organisms, the fish microbiome can be affected by MP, with presumed widespread effects on animal health [28,29,30,31,32,33]. Indeed, gut dysbiosis has been repeatedly linked to various metabolic disorders [34,35,36,37]. 

Finally, the combined effect of oxidative stress, inflammation, and gut dysbiosis could have an even more pronounced impact on fish during early development when energy usage and physiological stress is high [38,39,40]. In particular, increased energy expenditure could negatively impact vital processes, such as tissue maintenance, locomotion, and growth of the individual. MP can also indirectly affect energy budgets by promoting an immune response to purge and remove plastic particles via inflammation [41].

In order to investigate the potential negative effects of MP on a freshwater fish model organism, we exposed larval zebrafish to polyethylene MP (10–40 μm) at a concentration of 20 mg/L. The range selected simulates a mixed-size exposure while providing an adequate representation of the type of microplastics found in the environment, and provides consistency with previous laboratory investigations using supra-ecological concentrations [42,43,44,45]. The experiment examined the acute and prolonged effects of MP exposure throughout early development by assessing oxygen consumption rates (OCRs), directed gene expression analysis, and microbiome diversity to assess the various impacts of differential MP exposure. Our results show that MP exposure during early life has no impact on metabolic or immunological systems but appears to have localized effects within the gut by increasing oxidative stress and disturbing the gut microbiome.

## 2. Methods 

### 2.1. Caretaking

Commercially obtained adult zebrafish (*Danio rerio*) were maintained in an aquatic housing system (Aquaneering, San Diego, CA, USA) with recirculating UV-treated de-chlorinated water maintained at 27 °C with a 14:10 h light dark cycle. Adult animals were fed twice daily with Adult Zebrafish Complete Diet (Zeigler, Gardners, PA, USA) and once with *Artemia naupii*. Adult fish were bred in tanks using standard procedures [46], and embryos were collected before being washed twice in 0.05% bleach solution and transferred to 90 × 15 mm petri plates filled with E3 buffer solution (5 mM NaCl, 0.17 mM KCl, 0.33 mM CaCl2, 0.33 mM MgSO4, 0.0006 mM methylene blue). The fish were then kept at 28 °C until day 5 post-fertilization (5 dpf), with E3 medium being replaced once every 24 h. At 5 dpf, the fish were transferred into 300 mL beakers and maintained in de-chlorinated control water or water containing MP. The MP fish were exposed to commercially obtained 10–45 µm polyethylene microspheres (Cospheric, Goleta, CA, USA) at a concentration of 20 mg/L. All fish (MP and control) were kept under these conditions for either 4 or 10 days, corresponding to 9 dpf and 15 dpf, respectively. Under similar experimental conditions, this treatment previously yielded consistent ingestion and accumulation of MP in all zebrafish larvae [13]. All treatments were maintained in a 27 °C water bath for the duration of the experiment. Daily, larvae were fed ad libitum Larval AP100 feed (Ziegler, Gardners, PA, USA), and approximately one third of the water, 100 mL of either MP or pristine freshwater, was replaced to maintain water quality and remove deceased larvae. As with prior work [13], mortality rates remained low (<2%) throughout the experiment. Bioassays were run in parallel using fish from 6 independent breeding events. All animal experimentations conform to the guidelines of the Canadian Council on Animal Care and were approved by the Brandon University Animal Care Committee on July 12 2016 under the research protocol 2016R03.

### 2.2. Respirometry 

At 9 and 15 dpf, the oxygen consumption rates of zebrafish larvae were measured using a fibre-optic respirometry system (OXY-4 mini, PreSens, Regensburg, Germany). Both oxygen consumption and temperature values were recorded in parallel with the AutoResp software v2.1.2 (Loligo^®^ Systems, Viborg, Denmark), and data were analyzed using RespR v1.1.0.0 [47]. The respirometry procedure was conducted in a temperature-controlled water bath (26.91 °C ± 0.02) for the duration of the experiment. Fish were placed into borosilicate chambers (2–3 mL) fitted with small optodes using aquarium-grade silicon (Marineland, Blacksburg, VA, USA). Magnetic stir bars allowed for adequate oxygen circulation and their consistent rotation was improved by the addition of rubber rings at the bottom of the tube. The system was calibrated with 100% O_2_ saturated E3 medium or dissolved sodium sulfite (0.02 g/mL solution). The fish were acclimated to the borosilicate chambers for 30 min and half of the total volume was replaced with fresh E3 prior to data collection. Each trial consisted of a 20 min sampling period featuring an early section (2 min), an intermediate section (8 min), and a late section (10 min). A sample size of 6 replicates (where each replicate included 5–10 fish depending on the size of the tube used) was used to determine OCRs between the treatments. We also measured background OCR (with no fish in chambers) and found these values to be negligible compared to larval OCRs. The intermediate sections were plotted using RespR to identify the most linear portion of the slope used for OCR calculation [47]. The OCRs were converted to nanomol/fish/hour to remain consistent with previous literature [13,48].

### 2.3. RNA Extraction, Reverse Transcription (RT), and qPCR

Following 4 or 10 days of exposure (9 dpf or 15 dpf), the larvae were anesthetized in 100 mg/L tricaine methanesulfonate (MS–222, Sigma-Aldrich, Darmstadt, Germany) and quick-frozen in liquid nitrogen before being stored at −80 °C until further manipulation. RNA was isolated from a total of 36 samples, comprising 9 replicates for control and MP treatments at both 9 and 15 dpf, using an RNeasy Mini extraction kit in accordance to the manufacturer’s protocol (RNeasy, Qiagen, Aarhus, Denmark). A final volume of 20 µL was obtained for each sample, and the quantity and quality of the RNA was determined by the use of a NP80 NanoPhotometer^®^ (Implen, Munich, Germany). Optical density ratios of 260/280 nm above 1.9 and concentrations above 500 ng/µL were targeted in order to provide a total of 2 μg of RNA for reverse transcription using the GoScript™ Reverse Transcription System (Promega, Madison, WI, USA). The transcribed cDNA was then diluted (1:10) prior to qPCR, as described before [49].

### 2.4. qPCR 

Nine genes were selected in order to examine the inflammatory response of larval zebrafish. Primer sets were obtained from previous publications or custom designed with the use of Primer3 software (Table 1, [50,51,52,53,54,55,56,57]). Primer specificity was confirmed by examining the top e-value in BLASTn (NCBI) and single product amplification confirmed by melt analysis of all reactions.

Gene expression analysis was carried by qPCR amplification using the QuantiNova™ SYBR Green PCR kit (Qiagen, Aarhus, Denmark), with a final volume of 15 µL per reaction (7.5 µL 2X SYBR Green PCR Master Mix, 0.7 µM forward and reverse primer, respectively, and 1 µL cDNA). Standard curves were created for each of the primer sets and the efficiency of each primer set was computed using Quantinova Rotor-Gene Q Series Software (Qiagen). Melting curve analysis was also performed to ensure single product amplification in each reaction. Only primer sets with suitable efficiency values (1.00 +/− 0.20) and single amplicon were used for further testing. The cycle threshold (Ct) values were automatically calculated using Rotor-Gene software using the corresponding standard curve. Relative gene expression levels were calculated using the ΔΔCt method [58], using the geometric mean of two housekeeping genes (*eF1α* and *Rpl-13a*) as a reference [49,58]. 

### 2.5. Respirometry and Gene Expression Statistical Analysis

Respirometry data and gene expression data were analysed in SigmaPlot software (SigmaPlot 14.0, Systat Software, San Jose, CA, USA). A two-way ANOVA was used to compare both the respirometry and gene expression data. All tests were performed at a significance level of *p* = 0.05, after passing equal variance and normality tests. 

### 2.6. Microbiome Analysis

#### 2.6.1. Sampling, DNA Extraction, Sequencing, and Pre-Processing

At both 9 and 15 dpf, 6 replicate samples of each treatment containing 20 individual zebrafish larvae each were anesthetized in 100 mg/L tricaine methanesulfonate, thoroughly washed with 100% ethanol, flash frozen, and stored at −80 °C until further analyses. For each sample, genomic DNA was isolated using the One-4-All Genomic DNA Miniprep Kit (BioBasic, Markham, ON, Canada).

Samples were then sent to Génome Québec Innovation Centre (McGill University, Montreal, QC, Canada) for quality control, PCR validation and reaction, amplicon barcoding, and normalization. The 16S primers used were 515F: GTGCCAGCMGCCGCGGTAA and 806R: GGACTACHVGGGTWTCTAAT [59]. A BioAnalyzer 2100 (Agilent Technologies, Santa Clara, CA, USA) was used to check the quality of amplicon libraries that were subsequently sequenced on the MiSeq platform in a paired-end 300 bp fashion. Raw sequence reads were deposited in the NCBI short sequence read archive (SRA) under the accession number PRJNA643286. The amplicons were adapter and quality trimmed in CLC Genomics Workbench v11.0.1 (Qiagen, Aarhus, Denmark; Quality Limit = 0.00316, Minimum Length = 15, default parameters herein). The reads were then processed in CLC v11.0.1 for downstream analyses, as described previously [60]. 

#### 2.6.2. CLC Analysis

We used the CLC Microbial Genomics Module 3.5 (Qiagen) pipeline for 16S analysis. First, operational taxonomic units (OTUs) were clustered at 97% similarity against the reference SILVA 132 database [61]. Chimeric sequences as well as OTUs detected in less than 0.05% of the total reads were then filtered out. 

Bacterial communities were compared across ages and between control and MP fish. The OTU table was rarefied before OTU alpha diversity analysis was carried out using Shannon and Simpson’s alpha diversity indices as well as total OTU in CLC. Differences between all groups were assessed with a non-parametric Kruskall–Wallis test followed by Mann–Whitney U pairwise tests (α = 0.05). 

Furthermore, beta diversity was evaluated with weighted and unweighted UNIFRAC as well as the Bray–Curtis index. Principal coordinates analyses (PCoA) of the distance plots were visualized, and multivariate analyses (PERMANOVA) were used to compare treatment differences. Differential abundance was computed using a binomial general linear model framework, and a likelihood test was used to detect differences across groups, followed by a Wald test for pairwise comparisons (FDR < 0.05).

## 3. Results

### 3.1. Respirometry

**Figure 1:**
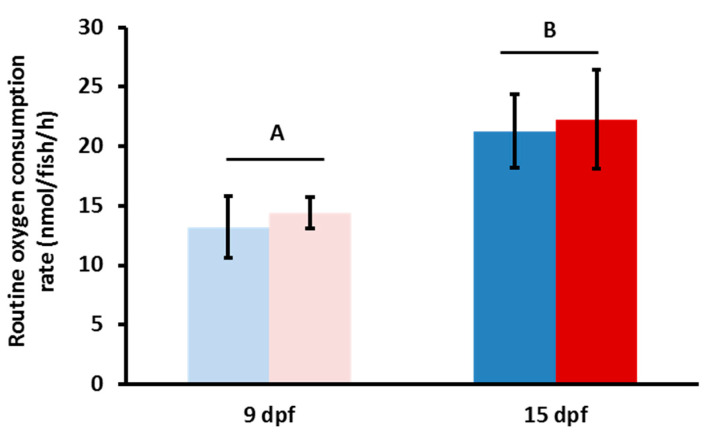
Oxygen consumption rates (OCRs) for microplastic (MP) exposed zebrafish. OCRs of MP fish after 5 days (pink) and 10 days (red) of exposure to MP and the respective age matched control fish (light blue/dark blue). No statistical significance was observed between the MP and control groups; however, there was a significant difference between time points (*n* = 6, *p* = 0.015).

We explored the effects of MP exposure on routine metabolic rate using closed-system respirometry (Figure 1). Mean oxygen consumption rates (OCRs) between fish raised in clear water (control) and MP-exposed fish at both developmental time points were not statistically significant (Figure 1). However, we were able to identify a difference in OCRs when comparing the two time points, 9 dpf and 15 dpf (*p* = 0.015). To test whether the developmental time point of the zebrafish was more important than the overall MP duration, we also examined a 10–15 dpf exposure period to MP; however, no significant difference was found when compared to the 5–15 dpf treatment (data not shown). Ultimately, no statistically significant difference in mean OCRs was identified in any of the treatments in regard to MP exposure (Figure 1). 

### 3.2. Gene Expression Analysis

**Figure 2:**
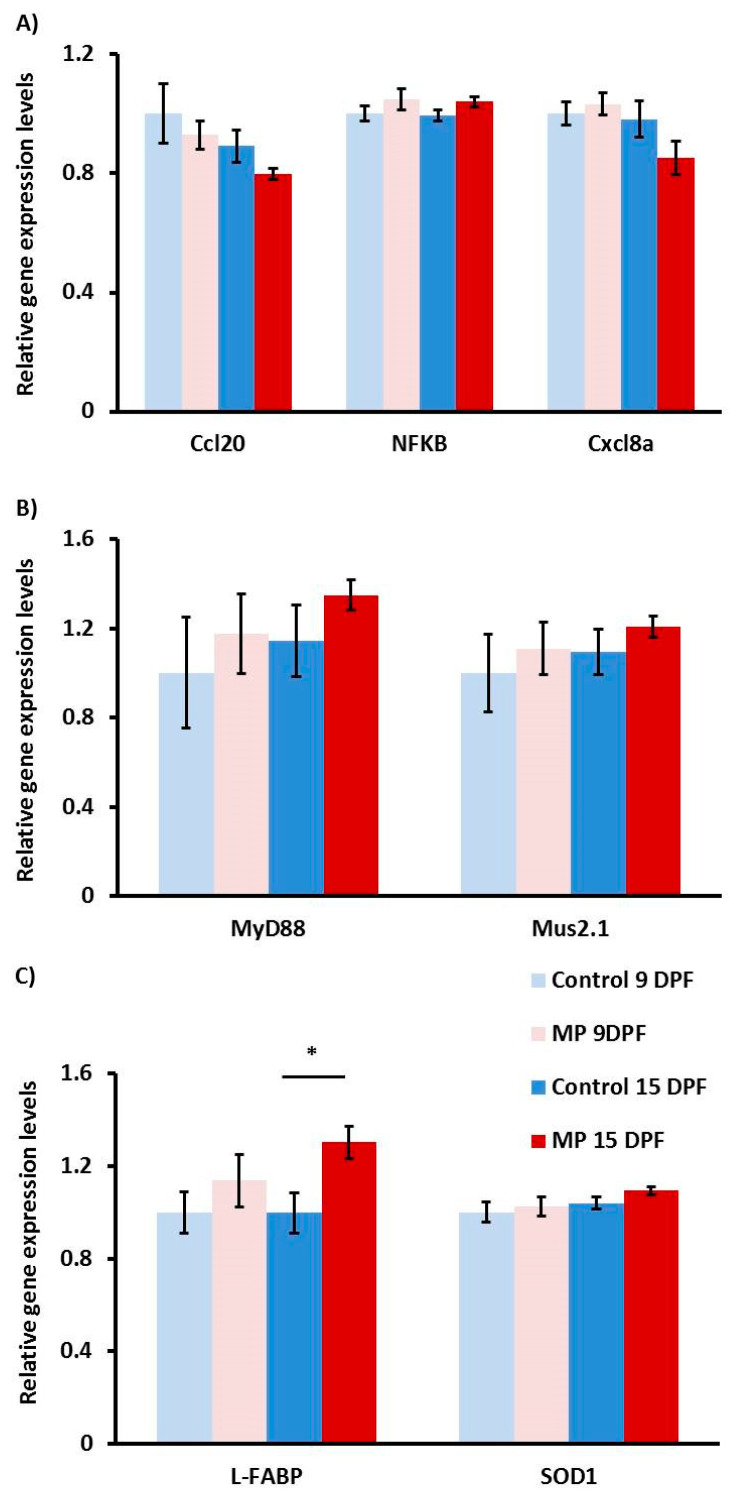
Gene expression in MP and control fish at 9 and 15 dpf. Gene expression analysis of inflammatory (**A**), microbial (**B**), and oxidative stress (**C**) genes in fish exposed to MP for 4 (pink) and 10 days (red) or aged matched control fish (light and dark blue). *EF1α*/*RLP-13a* were used as housekeeping genes. Statistically significant differences are indicated with an asterisk (*n* = 9, *p* < 0.05).

In order to assess the effects of MP exposure on various cellular response pathways, we used qPCR and evaluated patterns of gene expression of nine gene targets involved with the inflammatory response, the gut microbiome, or oxidative stress (Figure 2). In terms of MP inflammatory response, none of the target genes (*ccl20*, *cxcl8a*, and *NF-κβ*) appeared to be affected across treatments. Although *ccl20* and *cxcl8a* both show a marginal reduction in gene expression in 15 dpf MP fish, this effect was not statistically significant (Figure 2A). Similarly, genes associated with the gut microbiome (*MyD88* and *Mus2.1*) exhibited relatively high variability resulting in no appreciable difference between treatments (Figure 2B). Finally, genes that are typically upregulated in periods of oxidative stress, *L-FABP* and *SOD1*, were also examined. In this case, we detected a significant increase in *L-FABP* expression in MP fish at day 15 (*p* = 0.022), but a marginal and insignificant increase in *SOD1* in the same fish (Figure 2C).

### 3.3. Structure of Gut Bacteria Communities

We aimed to characterize the effect of MP exposure on the microbiome of zebrafish larvae for different exposure duration. Fish were exposed to MP or control conditions and sampled at 4 and 10 days, and their DNA was extracted (*n* = 6 for each treatment and time point) and was subjected to high throughput 16S sequencing. We obtained a total of 3,535,330 reads, of which 42% were retained as OTU reads after quality trimming and chimeric read removal. CLC analysis assigned these reads to 1239 OTUs at >97% similarity, and after selecting OTUs present in over 0.05% (*n* = 105) of the reads, these were identified from 7 phyla, 9 classes, 24 orders, 33 families, and 62 genera (see Supplementary File 1). 

### 3.4. Core Microbial Composition

**Figure 3:**
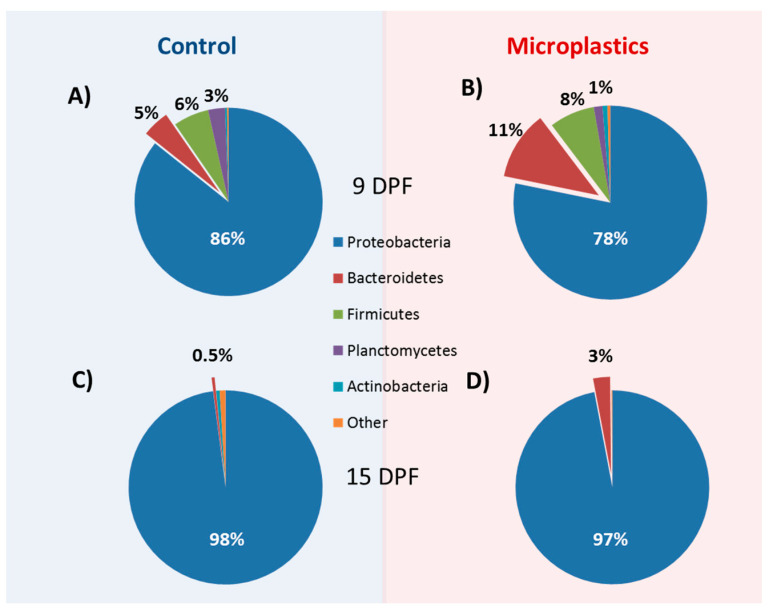
Relative phylum composition of control and MP-exposed zebrafish larvae. Zebrafish larvae were exposed to control conditions (**A**,**C**) and microplastics (**B**,**D**) for 4 days (**A**,**B**) and 10 days (**C**,**D**), respectively. Percentages represent the relative abundance of the most represented phyla.

In this study, we describe the core microbiome as taxa that are present in all 24 of the samples sequenced, regardless of treatment and time point. At the phylum level, *Proteobacteria* had a consistent dominating presence as the most abundant phylum (90%) across all samples and treatments. Indeed, the next most abundant ubiquitous phyla, *Bacteroidetes* and *Cyanobacteria*, only each averaged 4% of the total reads in all samples (Figure 3). At the genus level, *Aeromonas*, *Pseudomonas*, and *Vibrio* were overall the most abundant and were among the 17 genera identified as the core microbiome common (Supplementary File 1). Thus, while there were some marked differences in microbial composition across samples and treatments (see below), there was a relatively strong presence of two major phyla and several genera in the larval zebrafish microbiome. 

### 3.5. Microbial Community Changes through Ontogeny

**Figure 4:**
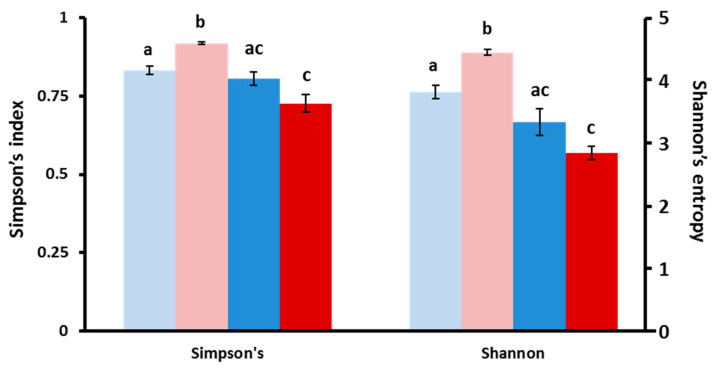
The effect of MP exposure on diversity indices in larval zebrafish. Simpson and Shannon OTU diversity indices obtained through CLC analysis for 9 dpf control (light blue) and microplastics (pink), as well as 15 dpf control (dark blue) and microplastics (red). Significant differences are indicated by different letters (*p* = 0.05).

In order to assess the differences in microbiota across treatments, we assessed the relative diversity of the samples. First, we normalized for sequencing depth and rarified all samples to 37,895 reads to examine changes in community structure. Overall, as the fish aged, we saw a relative decrease in community diversity (Figure 4). Although the general pattern applies to both MP and control treatments, it was only statistically significant for all three indicators used in MP fish (observed OTUs, Shannon and Simpson indices), while only the total observed OTU index was significantly decreased in older control fish. However, when we delve into the specifics, we see the number of ubiquitous phyla decreasing from five to only two, *Bacteriodetes* and *Proteobacteria*, during the 9 to 15 dpf transition (Supplementary File 1). Incidentally, the abundance of these two phyla increased with the age of the fish, while others, such as *Firmicutes* and *Planctomycetes*, were reduced in older fish. These trends were further confirmed as several genera of the *Burkholderiaceae* and *Comamonadaceae* family, for example, markedly decreased in older fish (Supplementary File 1). Overall, there was strong evidence of an ontogenic shift in microbial fauna in the developing fish. 

### 3.6. MP Exposure Disturbs the Microbiome 

While the age of the fish appears to have an influence on the microbiota composition within each treatment group, we were mostly interested in the differences within and between treatments. We compared beta diversity differences among our samples using permutational multivariate analysis (PERMANOVA). Overall, microbial communities were different across treatments/exposures (PERMANOVA: F = 6.16, *p* = 3 × 10^−5^) and particularly according to the age of the fish (PERMANOVA: *p* = 3 × 10^−5^). However, there was no overall significant impact of MP treatment compared to control when combining both time points (PERMANOVA: F = 1.11, *p* = 0.342). When examined within age groups, only 9 dpf fish showed a significant difference in bacterial communities in MP-treated fish compared to controls (*p* = 0.013). However, at both time points, there was an increased abundance of *Bacteroidetes* in MP fish, as well as changes in the abundance of several genera (Supplementary File 1). This overall suggests that, while larval zebrafish undergo broad ontogenic microbial changes over the exposure period, there is an evident effect of MP on the microbiome of larval fish, particularly during early development.

## 4. Discussion

Plastic pollution is quickly becoming one of the most concerning forms of anthropogenic pollution, with numerous studies indicating increased levels of MP in our water systems [62,63,64]. MP can accumulate inside aquatic species, rapidly leading to bioaccumulation and homeostatic imbalances [65,66]. For example, wild fish are likely encountering MP chronically and thus serve as excellent models for the study of MP toxicity in a living system [67]. A number of studies have demonstrated that MP pollution is indeed harmful, even under acute exposures, with polystyrene MP causing gut dysbiosis, inflammation, oxidative stress, and impaired swimming performance in adult zebrafish [41,68]. However, exposure to polyethylene MP has shown conflicting results in adult zebrafish, with some suggesting that polyethylene MP pose little to no harm [69], while others describe signs of oxidative stress and inflammation [16]. Here, we present additional evidence that there is a limited impact of acute MP exposure on developing larval zebrafish. Indeed, ten days of exposure to supra-ecological concentrations of MP does not seem to affect the overall metabolism of the fish and has a limited impact on inflammatory molecular responses, but it seems to trigger an elevated ROS response in the larvae and a concurrent dysbiosis of the larval microbiome. 

In order to assess the effect of MP ingestion at the whole organism level, we monitored larval oxygen consumption in a closed respirometry system. Indeed, changes in oxygen consumption could provide an indication of changes in the metabolic status of the animal, including changes in immunological activity and physiological stress [70,71]. Overall, larval oxygen consumption rates were in line with previous studies, with an evident increase in oxygen consumption as the larvae ages [48]. However, we were unable to detect any differences in oxygen consumption between age-matched control and MP-exposed fish, suggesting no global effects on the larval metabolic function. Increased OCRs may indicate the presence of physiological stressors such as widespread inflammation or decreased nutrient absorption [72]. Although similar oxygen consumption rates could indicate a lack of response, we cannot rule out a localized immune response to MP in the fish which may not have broad metabolic effects [13,44]. To further investigate this possibility, we examined the expression of several immune response genes following distinct durations of MP exposure.

Early on in larval zebrafish development, the immune system relies entirely on the innate response for protection [73]. Though efficient, this system requires activation via several pattern recognition receptors that recognize specific exogenous molecules and trigger the release of inflammatory cytokines [22]. We hypothesized that these receptors could be activated by polyethylene particles and thus decided to examine the expression of two genes involved in the broad spectrum *MyD88*-dependent pathway. *MyD88* is largely responsible for the signal transmission of toll-like receptors associated with inflammation, ultimately upregulating cytokine *NF-Kβ* [74,75,76]. We were unable to find any significant difference in *MyD88* or *NF-Kβ* expression between either of the time points and treatments, suggesting an absence of generalized inflammation via this pathway. However, it is possible that, given the broad tissue distribution of these markers, our approach measuring whole body gene expression levels may mask small localized changes in intestinal tissues. Considering this caveat, we investigated two cytokines (*ccl20* and *cxcl8a*) closely associated with the gut region to provide a more targeted assessment. Both cytokines have been associated with localized gut inflammation in zebrafish in various contexts, which may identify them as key contributors to gut inflammation [77,78]. However, once again, we did not detect any differences in gene expression, further suggesting that intestinal inflammation does not occur in larval zebrafish exposed to MP.

Despite the apparent lack of inflammation, we suspected that MP in the gut could lead to increased oxidative stress resulting from physical abrasion of enterocytes. Therefore, we examined the transcript accumulation of two intestinal antioxidants found in the intestine of larval zebrafish: liver fatty-acid binding protein (*L-FABP*) and superoxide dismutase 1 (*SOD1*). *L-FABP* mitigates hepatic and intestinal ROS production by binding to fatty acid metabolites and heme ligands [79]. Similarly, *SOD1* limits ROS using zinc and copper ions, producing non-reactive hydrogen and oxygen atoms [80]. We observed an increase in *L-FABP* expression during the MP 9–15 dpf time period; however, there was no observable difference in *SOD1* expression, possibly due to the wide expression of *SOD1* in contrast to a more tissue-specific expression of *L-FABP*. Together, as previously suggested, our results suggest that MP exposure promotes increased ROS in the zebrafish gut [16,28,81]. 

### Microbiome

The gut of the zebrafish is quickly colonized by microorganisms during the early phases of larval development following the completion of the intestinal tract, resulting in a microbial community known as the “microbiome”. The microbiome is primarily colonized by bacteria that originate from the chorion of the larvae and the surrounding environment during the initial feeding event [82]. Consequently, variability in environmental conditions has made it complex to assess the composition of a core intestinal microbiome in zebrafish, particularly when considering the rapid development of these fish and the variability among fish facilities and strains [83,84]. With these aspects in mind, we ensured that zebrafish larvae were treated equally during our two periods of MP exposure (5–9 dpf and 5–15 dpf) to identify potential time-sensitive responses to polyethylene MP.

As the fish aged, the diversity of the microbiome was significantly reduced. Furthermore, trends of dominant genera changed from 9 dpf to 15 dpf in both the absence and presence of MP. In all fish, dominant genera such as *Variovorax* increased with age, while others such as *Rheinheimera* disappeared. When comparing the endpoints of both treatments, we were able to identify a decrease in *Aeromonas* sp. and an increase in *Vibrio* sp., a change which may be indicative of natural microbiome development [85]. Increased microbiome diversity is expected to occur at approximately 10 dpf as the zebrafish larvae transition from larvae to juveniles, meaning that this period of development is naturally highly dynamic, even in the absence of MP or other contaminants [84]. We were able to detect two distinct microbial community profiles between half of our samples in each of the MP treatments, which suggests that our time points represent this transitional state and may have increased the susceptibility to dysbiosis via MP exposure.

Larval microbiota was overall more affected by MP at 9 dpf than 15 dpf, indicating that dysbiosis in response to this pollutant may be time- or life stage-sensitive. In contrast to older phases of development, larval zebrafish seem to experience greater gut microbial shifts due to environmental factors [84,86,87]. However, at the phylum level, MP induced an increase in *Bacteroidetes* at both life-stages and a decrease in *Actinobacteria* at 15 dpf, a combination that has been found in intestinal inflammation pathologies [88]. Furthermore, some members of the sub-genera Bacteroids have also had their presence associated with various human metabolic diseases such as obesity, autism, type II diabetes, and colorectal cancer [34,35,36,89]. Therefore, although MP exposure only transiently affects bacterial communities, these changes could negatively impact the health of the fish during these transitional life stages. 

Interestingly, 15 dpf MP larvae demonstrate concurrently a higher abundance of *Bacteriodetes* and an increase in ROS, as evidenced by an increase in *L-FABP* expression compared to control fish at 15 dpf. While we can only speculate regarding the interaction between ROS production and increased abundance of this bacterial phylum, it is possible that increased oxidative stress in MP fish promotes the growth of these bacteria. Indeed, while cells of many micro and macro-organisms are typically affected by ROS [68,90,91], some microbial species, including members of the *Bacteriodetes*, have effective defense mechanisms against damaging oxidative stress, which may give them a selective advantage over other micro-organisms [92,93,94]. Alternatively, it is possible that the proliferation of these bacteria may be the underlying cause of oxidative stress. Regardless of the proximate causal agent, we confirm that MP exposure is detrimental to larval zebrafish. 

## 5. Conclusions

MP exposure is an environmental concern, as virtually every single ecosystem is plagued with these stable microscopic particles. Here, we confirm that MP exposure negatively affects a freshwater organism as it can result in microbial dysbiosis and increased oxidative stress in larval zebrafish. These effects were asynchronous, with pronounced dysbiosis after only four days of exposure and a subsequent increase in ROS after longer exposure. Furthermore, we were unable to identify any determinants of metabolic distress or inflammation across three independent time points, which suggests that, during early development, MP toxicity may be localized (guts) and does not occur at the whole organism level. Our time course also suggests that the zebrafish gut is most susceptible to MP-induced dysbiosis following the transition from the larval to juvenile state and that, by promoting changes in the microbial communities, such as the proliferation of potentially pathogenic bacteria, MP has the potential to negatively affect fish health at a particularly vulnerable phase of development. Overall, our results corroborate the accumulating evidence that aquatic organisms are negatively affected by MP exposure, though the consequences at the tissue and whole organismal level remain complex and should certainly remain an active area of research.

## Figures and Tables

**Table 1:** List of primers used in qPCR assays of gene expression.

**Gene**	**Forward Primer**	**Reverse Primer**	**Accession Number**
*ef1α* ^51^	CTTCTCAGGCTGACTGTGC	CCGCTAGCATTACCCTCC	AY422992
*Rpl-13a* ^52^	TCTGGAGGACTGTAAGAGGTATGC	AGACGCACAATCTTGAGAGCAG	NM_212784.1
*MyD88* ^53^	GAGGCGATTCCAGTAACAGC	GAAAGCATCAAAGGTCTCAGGTG	NM_212814.2
*Ccl20* ^54^	ATCAATCTGCGCTAATCCATCAC	TGGTGAACATGCTCATCGTCTT	NM_001113595.1
*L-FABP* ^55^	ACGTGGCAGGTTTACGCTCAG	TTGGAGGTGATGGTGAAGTCG	BC164928.1
*SOD1* ^56^	GTCGTCTGGCTTGTGGAGTG	TGTCAGCGGGCTAGTGCTT	AY324390.1
*NF-κβ* ^56^	CCAAATCCCAAAAGGTTAGAGATTT	CCTCTTAGGGCTGAGCGAATT	XM_005156814.2
*Cxcl8a* ^57^	TGTTTTCCTGGCATTTCTGAC	TTTACAGTGTGGGCTTGGAGGG	XM_009306855.3
*Mus2.1* ^50^	TGGTGGACCAGTGTGAAAAA	GGTCCAAAACCCAGCTACAA	XM_021470771.1

## Supplementary Materials

The following are available online at https://www.mdpi.com/2305-6304/8/3/55/s1, file 1: Differential microbial abundance after 4 and 10 days of MP exposure in larval zebrafish.
